# Analysis of the Influencing Factors of Immunological Nonresponders in Wuhan, China

**DOI:** 10.1155/2022/5638396

**Published:** 2022-08-08

**Authors:** Enze Lei, Shuna Jin, Wei Ni, Manlin Feng, Yanhe Luo, Lianguo Ruan, Mingzhong Xiao, Jianzhong Liu

**Affiliations:** ^1^College of Traditional Chinese Medicine, Hubei University of Chinese Medicine, Wuhan, Hubei 430061, China; ^2^College of Basic Medicine, Hubei University of Chinese Medicine, Wuhan, Hubei 430061, China; ^3^Hubei Provincial Hospital of Traditional Chinese Medicine, Wuhan, Hubei 430061, China; ^4^Wuhan Jinyintan Hospital, Wuhan, Hubei 430061, China

## Abstract

**Objective:**

CD4^+^ cell recovery is hampered in some human immunodeficiency virus (HIV)-infected patients, despite a successful highly active antiretroviral therapy (HAART) with suppressed viral replication. We investigated the factors that might have hindered the CD4^+^ cell recovery in these patients.

**Methods:**

In this retrospective study, we collected the data of all immune nonresponders (INRs) in Wuhan, China, until the end of 2020. A linear model was constructed based on the data from 220 patients with baseline and follow-up records. The response variables in this study were the CD4^+^ cell count increase. The predictor variables considered in this study were those factors likely to affect the CD4^+^ cell recovery.

**Results:**

Our findings revealed that the plasma HIV-1 viral load of all patients was suppressed and 87.3% patients' CD4^+^ cells was increased after more than one year of the HAART treatment. In addition, their last follow-up showed a significant reduction in complications. In our results, the body mass index (BMI), number of months since HIV diagnosis to HAART start, and nonuse of co-trimoxazole were negatively correlated with the increase in CD4^+^ cells (*P* < 0.05). However, there were positive associations between serum creatinine levels and CD4^+^ cell recovery (*P* < 0.05). Further stratified analyses indicated that the associations between HAART replacement or creatinine usage and CD4^+^ cell growth were only observed in those participants with a BMI <18.5 (*P* < 0.05).

**Conclusions:**

An early initiation of HAART and co-trimoxazole preventive therapy (CPT) can promote immune reconstitution. BMI and serum creatinine can serve as monitoring indicators of immune reconstitution prognosis after the HAART.

## 1. Introduction

Highly active antiretroviral therapy (HAART) can suppress the human immunodeficiency virus (HIV) viral load to undetectable levels and increase the CD4^+^ cell counts. With increasing accessibility to HAART, the acquired immunodeficiency syndrome (AIDS)-related morbidity and mortality in HIV-1-infected individuals have sharply diminished [1, 2]. However, considering the heterogeneity among study populations and discrepancy in definitions, the optimal treatment could persistently suppress viral replication but fail to restore CD4^+^ cell counts in approximately 5–45% patients [3–6]. These patients are referred to as immunological nonresponders (INRs), and an impaired immunological response in them is linked to an increased risk of disease progression and death [4, 7]. Thus, various mechanisms of poor immune reconstitution in HIV-1-infected patients were intensively studied and specific therapeutic strategies to restore immunity were continuously considered [8].

China still faces the challenge of HIV/AIDS. In 2020, the annual HIV incidence reached 4.4/100,000, resulting in 62,000 new HIV/AIDS infections. In addition, 18,000 people died from AIDS, which is ranked first among Class B infectious diseases [9]. Wuhan is the capital of Hubei province and the central city of Central China. In 2020, the city was estimated to cover an area of 8,569.15 square kilometers, with a permanent population of 12.447 million [10]. At the end of December 2020, Wuhan Jinyintan Hospital was still treating 6,088 AIDS patients from Wuhan. As reported elsewhere [8], there were many HIV-1-infected patients with low CD4^+^ cell counts, despite the suppression of viral replication after the HAART.

INRs present severe immune dysfunction and the CD4^+^ cell count change is the optimal reference index in the prognosis of immune reconstitution [11–13]. At present, the value of 200 CD4^+^ cells/ml is considered as a critical threshold under which the immune reconstitution fails [3, 6]. Several studies have been carried out with regard to the CD4^+^ cell recovery, but articles that analyze the influencing factors in a particular area of the population are insufficient. Therefore, we have attempted to analyze the factors contributing to poor immune reconstitution—by comparing patients' baseline and follow-up status based on the reported risk factors [14–18] and data collected in this study.

## 2. Materials and Methods

### 2.1. Patients and Study Design

This was a retrospective study; all the event exposure had already occurred in the past. The research data were collected from China's Disease Prevention and Control Information System—Basic Information System for AIDS Prevention and Control. We collected data on patients with HIV/AIDS who visited and registered at Wuhan Jinyintan Hospital from October 2009 to December 2020. By the end of 2020, 5,464 patients underwent the HAART for more than 12 months in the Wuhan Jinyintan Hospital. The total number of INRs was 222, which accounts for 4.1% of these cases. According to the Chinese reference standard, we enrolled INRs who fulfilled all the following requirements: (1) patients who were outpatients and received the HAART for more than 12 months, (2) patients who had sustained viral load suppression (<50 copies/ml), (3) patients who had CD4^+^ cell count <200 cells/ml during follow-up, and (4) patients who had baseline and follow-up records. This study used the time of HAART start as baseline, and the date of the last follow-up visit before December 31, 2020 was used as the follow-up endpoint. Excluding two patients with missing baseline CD4^+^ cell count, baseline and follow-up data (Additional file1: Table S1) of the rest of the 220 patients were ultimately included in the study. All participants provided written informed consent at enrollment, and the ethics protocol was approved by the Ethics Committee of Hubei Provincial Hospital of Traditional Chinese Medicine and Wuhan Jinyintan Hospital (HBZY2018−C23−02).

We analyzed the factors that contributed to poor immune reconstitution by comparing patients' baseline and follow-up status. The response variables in this study were the CD4^+^ cell count increase. Based on the reported risk factors [14–18] and data collected in this study, the predictor variables that seemed to affect the CD4^+^ cell counts were the following: gender, body mass index (BMI), age at follow-up, duration from HIV diagnosis to HAART start, duration of HAART, transmission route, WHO clinical stage, follow-up times, co-trimoxazole preventive therapy (CPT), HAART options, HAART replacement, creatinine, hyperlipidemia, liver function impairment, and anemia.

### 2.2. Statistical Analysis

The datasets were established by Microsoft Excel program, and then subjected to SPSS26.0 software for statistical analysis. The numerical data were expressed as frequencies and calculated by the *χ*^2^ test on a crosstabulation. For continuous variables, data were expressed as median (M) with interquartile range (IQR) (P25, P75) and beta (*β*) with a 95% confidence interval (CI). The comparison before and after HAART was performed by the Wilcoxon Signed Rank Test. The relationships between the collected influencing factors and CD4^+^ cell count increase were explored by Mann–Whitney and Kruskall–Wallis tests. Covariates were selected as potential confounders because they were reported to be associated with CD4^+^ cell count increase or *P* value <0.1 on the basis of the statistical consideration. Linear regression models were used to estimate the associations between covariates and CD4^+^ cell count increase. In addition, we also conducted stratified analyses by BMI (BMI < 18.5 kg/m^2^, BMI 18.5–25 kg/m^2^, and BMI ≥ 25 kg/m^2^) to explore the differences in the association between the screened influencing factors and CD4^+^ cell count increase among different BMI groups. Two-sided *P* value <0.05 was considered statistically significant.

## 3. Results

Table 1 lists the baseline characteristics of 220 patients included in the study. The median age of these patients was 49.4 years (IQR 33.0–59.4), and the male-to-female ratio was 8.6 : 1. The BMI <18.5 kg/m^2^ and BMI ranges 18.5–25.0 kg/m^2^ groups accounts for 19.5% and 69.5% of all patients, respectively. The routes of acquisition of HIV were injection drug abuse (1.4%), homosexual (44.1%), heterosexual (51.4%), and unknown in seven cases. In our study population, patients in stages I, II, III, and IV (WHO clinical stage) were 5.5%, 28.6%, 42.3%, and 23.6%, respectively. In addition, these patients received the HAART for the first time after baseline data entry and had following treatment in Wuhan Jinyintan Hospital.

The comparison of follow-up and baseline conditions of the patients is given in Table 2. In our study population, most patients started the HAART 1–3 months after HIV diagnosis, and the duration of HAART ranged from 1 to 11 years. 94.1% of patients received the HAART as the first-line treatment (3TC + TDF + EFV, 3TC + AZT + EFV, NVP+3TC + AZT); whereafter, 3.1% of the patients changed their treatment plan due to changes in their condition or financial situation. Co-trimoxazole was received at baseline in 156 patients; but after the HAART, the number increased to 195. The results indicated that the median number of HIV-1 viral load at baseline was 97,830 copies/ml (IQR 35,476–254,172). After the HAART, all HIV-infected patients achieved viral suppression (<50 copies/ml), and 72.3% of them had plasma HIV-1 virus levels below 20 copies/ml (*P* < 0.01). The median number of CD4^+^ cells was 39 cells/*μ*l at the baseline; this number increased to 162 cells/*μ*l at the follow-up (*P* < 0.01). Haemogram tests showed a significant increase in white blood cell (WBC) count, platelet (Plt) count, hemoglobin (Hb) count, triglyceride (TG) level, total cholesterol (TC) level, and a significant decrease in aspartate aminotransferase (AST) level, alanine aminotransferase (ALT) level, and total bilirubin (T.BIL) level (*P* < 0.05 for all). From the baseline to follow-up, the number of patients with liver function impairment decreased from 98% to 74%; those with leukopenia decreased from 91% to 46%; and those with anemia decreased from 78% to 24% (*P* < 0.01). In addition, the clinical comorbidities of the patients significantly reduced from the baseline to follow-up (*P* < 0.01 for all) (Additional file 1: Table S2).

After more than one year of the HAART treatment, 87.3% patients' CD4+ cells was increased but did not exceed 200 cells/*μ*l. By analyzing the relationship between the screened influencing factors and CD4+ cell count increase (Table 3) we found that the duration from HIV diagnosis to HAART start, co-trimoxazole at the baseline, HAART options at the baseline, and creatinine at follow-up were statistically significant (*P* < 0.05 for all). The median number of CD4^+^ cell count increase in the months since HIV diagnosis to HAART start <1 was 107.0 cells/*μ*l (IQR 55.0–148.0), while for the months since HIV diagnosis to HAART start 1–3 were 94.0 cells/*μ*l (IQR 51.0–132.0), and for the months since HIV diagnosis to HAART start >3 were 65.0 cells/*μ*l (IQR −48.5–127.0; *P* < 0.05 for all). Patients using co-trimoxazole had a higher CD4^+^ cell count increase than those who did not use it at the baseline (median, 105.0 vs. 60.5 cells/*μ*l; *P* < 0.01). The median number of CD4^+^ cell count increase at the baseline in the HAART (3TC + TDF + EFV) was 96.0 cells/*μ*l (IQR 55.5–133.0); in the HAART (3TC + AZT + EFV) was 38.0 cells/*μ*l (IQR −18.5–130.0); in the HAART (NVP + 3TC + AZT) was 73.5 cells/*μ*l (IQR −37.3–133.8); and in the HAART (Others) was 113.0 cells/*μ*l (IQR 97.5–152.0; *P* < 0.05). The CD4^+^ cell count increase in the group with low creatinine was lower than that of the group with high creatinine at the follow-up (median, 83.0 vs. 113.0 cells/*μ*l; *P* < 0.01).

Table 4 displays the linear relationships of CD4^+^ cell count increase and the characteristics of the study participants. We found that the BMI of the participants, duration from HIV diagnosis to HAART start, WHO clinical stage, co-trimoxazole at the baseline, and creatinine at the follow-up were associated with the CD4^+^ cell count increase in univariate linear analysis (*P* < 0.05 for all). When factors including the gender, BMI, age at follow-up, duration from HIV diagnosis to HAART start, duration of HAART, transmission route, WHO clinical stage, co-trimoxazole and HAART options at baseline, HAART replacement, and creatinine at follow-up were included in the multivariate model, there were still significant CD4^+^ cell count increase associations with the participants' BMI, duration from HIV diagnosis to HAART start, co-trimoxazole at the baseline, and creatinine at the follow-up (for BMI, *β* = −4.2, 95% CI: −8.1 to −0.2; for duration from HIV diagnosis to HAART start, *β* = −17.0, 95% CI: −33.4 to −0.5; for co-trimoxazole at baseline, *β* = −98.5, 95% CI: −129.3 to −67.7; for creatinine at the follow-up, *β* = 0.9, 95% CI: 0.1 to 1.7; *P* < 0.05).

The stratified analyses by BMI are shown in Table 5. In the multivariate linear regression models, co-trimoxazole at the baseline was the only significant factor regarding the CD4^+^ cell count increase in the participants with BMI 18.5–25.0 kg/m^2^ and BMI ≥25.0 kg/m^2^ (for BMI 18.5–25.0 kg/m^2^, *β* = −82.0, 95% CI: −114.9 to −49.1; for BMI ≥25.0 kg/m^2^, *β* = −595.7, 95% CI: −756.2 to −435.2; *P* < 0.05 for all). Whereas in the participants with BMI <18.5, the associations between co-trimoxazole at the baseline, HAART replacement or creatinine at the follow-up and the CD4^+^ cell count increase were significant (for co-trimoxazole at the baseline, *β* = −93.7, 95% CI: −157.8 to 29.5; for HAART replacement, *β* = −54.2, 95% CI: −101.2 to −7.2; for creatinine at the follow-up, *β* = 1.5, 95% CI: 0.1 to 3.0; *P* < 0.05 for all).

## 4. Discussion

INRs are HIV-infected patients with a suppressed viral load but with only a suboptimal increase in CD4^+^ cell count after the HAART initiation. Our findings revealed that the plasma HIV-1 viral loads were suppressed, and the CD4^+^ cell counts increased 87.3%, on average in all the patients, after more than one year of the HAART treatment. This result was in concordance with a study carried out by Valdez et al. [19]. In addition, their last follow-up showed a significant reduction in complications, although the CD4^+^ cell count of these patients was less than 200. This finding was unexpected because a higher incidence of opportunistic infections and tumors in INRs was revealed in several studies [7, 11, 13]. We speculate that this may be related to the long-term, regular use of the HAART and anti-infection care and treatment.

In previous studies, hemoglobin, white blood cell, triglyceride, or cholesterol was thought to be related to immune rebuilds in AIDS patients [20–22]. These INRs showed statistically significant changes in these blood indicators (Table 2). However, when we further explored their relationship with CD4^+^ cell count increase, only a linear relationship was found between the serum creatinine and CD4^+^ cell count increase (Table 3), whereas a positive correlation between the creatinine levels and CD4^+^ cell count increase was observed in participants with a BMI <18.5 kg/m^2^.

There was evidence showing that increasing age, male heterosexuality, and injection drug use were risk factors for not achieving a CD4^+^ count >200 cells/*μ*l [14]. The CD4+ cell count recovery varied mostly with the duration of ART and gender [23, 24]. The late WHO clinical stage has a significant negative impact on the CD4^+^ cell count and survival time [15, 25]. The above studies analyzed the influencing factors among infected persons with different immune reconstitution statuses. In this study based on patients with poor immune reconstitution, the above factors were not found to be related to the CD4^+^ cell count increase.

Motayo et al. [16] showed that the earlier the HAART starts, the faster the virus levels fall and the immune system rebuilds. Our results were consistent with these reports. Duration from HIV diagnosis to HAART start was negatively correlated with the CD4+ cell count increase. Koethe et al. [17] demonstrated that the CD4^+^ cell count increase was negatively correlated with BMI at the stage of 18.5 to 22 kg/m^2^. However, a recent single-cohort study of HIV-infected adults in the southeast of the USA (a region with a high prevalence of obesity) found that a 12-month CD4^+^ lymphocyte gain after ART initiation was greatest among those with a pretreatment BMI of 25 to 30 kg/m^2^, and it diminished above and below this range [26]. In our results, patients with a BMI in 18.5–25.0 kg/m^2^ accounts for 69.5% of all patients. In this group, BMI was negatively correlated with the CD4^+^ cell count increase. The finding that greater adipose tissue is associated with peripheral CD4^+^ cell recovery on HAART should be explored further in translational studies to understand the mechanisms and potential therapeutic implications.

A study of the HAART options among treatment-naive patients did not exhibit a significant difference in immune recovery after 48 weeks between lopinavir/ritonavir and efavirenz as initial therapies. Other studies in patients with CD4^+^ T lymphocytes <100 cells/*μ*l demonstrated that efavirenz-induced immune reconstitution was not inferior to that induced by boosted protease inhibitors (PIs) [27]. In the weighted intent-to-treat analysis, patients spent an average of 74 (95% confidence interval: 41, 106) additional days alive, with a suppressed viral load on the raltegravir regimen than on the efavirenz regimen over 2.5 years of the study period. The CD4^+^ cell recovery was also superior under the raltegravir regimen [18]. However, in our study, there was no statistical difference in the increment of CD4^+^ cells in either the first-line program or program change in China.

Daily co-trimoxazole is recommended for African adults living with HIV, irrespective of antiretroviral treatment, immune status, or disease stage [28]. The CD4^+^ cell count test, early identification and treatment of AIDS patients, together with co-trimoxazole prevention and treatment programs were important approaches in extending the survival time and reducing the death rates from AIDS-related illnesses [29]. Our study showed that the increment of CD4^+^ cells in HIV patients who took co-trimoxazole was significantly higher than that in those who did not take it, which was just consistent with the above research results. This association was significant across the different BMI subgroups.

There are some limitations to this study that should also be taken into account. First, because of the cross-sectional nature of our study, we could not consider the continuous changes of related indicators. Next, further research is necessary to extrapolate our findings to immunological responders.

## 5. Conclusions

Altogether, an early initiation of the HAART and CPT can promote immune reconstitution. BMI and serum creatinine may serve as monitoring indicators of immune reconstitution prognosis after the HAART.

## Figures and Tables

**Table 1:** Baseline characteristics of the study patients (*N* = 220).

Characteristics	*N* (%) or median (IQR)
Age (years)	49.4 (33.0, 59.4)
<29	33 (15.0%)
29–39	41 (18.6%)
39–49	34 (15.5%)
≥49	112 (50.9%)
Height (cm)	170.0 (165.0, 174.0)
Weight (kg)	59.0 (53.0, 66.0)
BMI (kg/m^2^)	20.8 (18.8, 22.5)
<18.5	43 (19.5%)
18.5–25.0	153(69.5%)
≥25.0	17 (7.7%)
Missing	7 (3.2%)

*Gender*	
Male	197 (89.5%)
Female	23 (10.5%)

*Transmission route*	
Drug use	3 (1.4%)
Homosexual	97 (44.1%)
Heterosexual	113 (51.4%)
Missing	7 (3.2%)

*WHO clinical stage*	
I	12 (5.5%)
II	63 (28.6%)
III	93 (42.3%)
IV	52 (23.6%)

**Table 2:** The comparison of follow-up and baseline conditions of the study patients.

Variable	Baseline	Follow-up	*P*
Years since HIV diagnosis	0.1 (0.1, 0.2)	3.3 (2.0, 5.7)	0.000
Years since HAART	—	3.0 (1.8, 5.0)	—
Follow-up times	—	15.0 (11.0, 22.0)	—
HAART options			0.000
3TC + TDF + EFV	153 (69.5%)	118 (53.6%)	
3TC + AZT + EFV	30 (13.6%)	27 (12.3%)	
NVP+3TC + AZT	24 (10.9%)	9 (4.1%)	
Others	13 (5.9%)	66 (30.0%)	
Co-trimoxazole			0.000
Yes	156 (70.9%)	195 (88.6%)	
No	64 (29.1%)	23 (10.5%)	
Missing	0 (0.0%)	2 (0.9%)	
Liver function impairment			0.000
Yes	98 (44.1%)	74 (33.3%)	
No	124 (55.9%)	125 (56.3%)	
Missing	0 (0.0%)	23 (10.4%)	
Leukopenia			0.000
Yes	91 (41.4%)	46 (20.9%)	
No	129 (58.6%)	150 (68.2%)	
Missing	0 (0.0%)	24 (10.9%)	
Anemia			0.000
Yes	78 (35.5%)	24 (10.9%)	
No	144 (64.5%)	174 (78.2%)	
Missing	0 (0.0%)	24 (10.9%)	
Viral load (copies/ml)	97830.0 (35476.0, 254172.0)	0.0 (0.0, 20.0)	0.000
CD4 counts (cells/*μ*l)	39.0 (20.3, 105.8)	162.0 (128.3, 183.0)	0.000
WBC count (×10^9^/L)	4.2 (3.3, 5.1)	4.8 (4.0, 5.7)	0.000
Plt count (×10^9^/L)	174.0 (135.8, 218.3)	205.5 (170.3, 241.5)	0.000
Hb count (g/L)	128.0 (113.0, 140.0)	144.0 (130.0, 153.0)	0.000
Cr (*μ*mol/L)	71.8 (61.0, 82.5)	70.0 (60.9, 82.0)	0.393
TG (mmol/L)	1.4 (0.9, 1.9)	1.5 (1.0, 2.4)	0.027
TC (mmol/L)	3.8 (3.3, 4.3)	4.4(3.8, 5.0)	0.000
FPG (mmol/L)	5.5(4.9, 6.4)	5.5 (5.2, 6.3)	0.159
AST (U/L)	28.0 (21.0, 37.0)	25.0 (20.0, 31.0)	0.000
ALT (U/L)	23.5 (14.0, 41.0)	20.0 (14.0, 28.5)	0.002
T.BIL (*μ*mol/L)	10.0 (7.5, 12.9)	7.5 (5.6, 10.7)	0.000

HAART, Highly active antiretroviral therapy; 3TC, Lamivudine; TDF, Tenofovir disoproxil; EFV, Efavirenz; AZT, Zidovudine; NVP, Nevirapine; WBC, White blood cell; Plt, Platelet; Hb, Hemoglobin; Cr, Creatinine; TG, Triglyceride; TC, Total cholesterol; FPG, Fasting plasma glucose; AST, Aspartate aminotransferase; ALT, Alanine aminotransferase; T.BIL, Total bilirubin. Data are presented as *N* (%) or Median (IQR). *P* values were determined by *χ*^2^ test or Wilcoxon signed rank test.

**Table 3:** The CD4^+^ cell count increase of different influencers.

Influencers		CD4^+^ cell count increase	*P*
Gender	Male (*N* = 197)	93.0 (39.0, 133.0)	0.716
	Female (*N* = 23)	89.0 (−7.0, 137.0)	
BMI (kg/m^2^)^a^	<18.5 (*N* = 43)	94.0 (26.0, 151.0)	0.151
	18.5–25.0 (*N* = 153)	96.0 (53.0, 133.5)	
	≥25.0 (*N* = 17)	56.0 (16.0, 98.0)	
	Missing (*N* = 7)	111.0 (−142.0, 166.0)	
Age at follow-up (years)^b^	<29 (*N* = 21)	96.0 (41.5, 128.5)	0.615
	29–39 (*N* = 40)	94.5 (25.3, 158.3)	
	39–49 (*N* = 30)	93.5 (−1.0, 155.0)	
	≥49 (*N* = 129)	92.0 (49.5, 130.0)	
Duration from HIV diagnosis to HAART start (months)^b^	<1 (*N* = 71)	107.0 (55.0, 148.0)	0.014
	1–3 (*N* = 101)	94.0 (51.0, 132.0)	
	≥3 (*N* = 48)	65.0 (−48.5, 127.0)	
Duration of HAART (months)^b^	12–24 (*N* = 73)	97.0 (57.5, 128.5)	0.172
	24–48 (*N* = 72)	100.0 (44.5, 146.8)	
	>48 (*N* = 75)	81.0 (12.0, 133.0)	
Transmission route	Drug use (*N* = 3)	—	0.420
	Homosexual (*N* = 97)	86.0 (27.0, 134.5)	
	Heterosexual (*N* = 113)	98.0 (55.0, 135.0)	
	Missing (*N* = 7)	57.0 (−12.0, 123.0)	
WHO clinical stage^a^	I (*N* = 12)	53.0 (−59.0, 114.5)	0.078
	II (*N* = 63)	86.0 (25.0, 129.0)	
	III (*N* = 93)	89.0 (42.0, 149.5)	
	IV (*N* = 52)	108.5 (74.8, 133.0)	
Follow-up times	≤10 (*N* = 53)	92.0 (32.5, 131.0)	0.782
	10–20 (*N* = 101)	98.0 (48.0, 133.5)	
	>20 (*N* = 66)	90.0 (19.5, 137.0)	
Co-trimoxazole^a^	Yes (*N* = 156)	105.0 (62.5, 137.8)	0.000
	No (*N* = 64)	60.5 (−68.3, 122.0)	
Co-trimoxazole^b^	Yes (*N* = 195)	93.0 (42.0, 133.0)	0.510
	No (*N* = 23)	96.0 (7.0, 164.0)	
	Missing (*N* = 2)	—	
HAART options^a^	3TC + TDF + EFV (*N* = 153)	96.0 (55.5, 133.0)	0.035
	3TC + AZT + EFV (*N* = 30)	38.0 (−18.5, 130.0)	
	NVP+3TC + AZT (*N* = 24)	73.5 (−37.3, 133.8)	
	Others (*N* = 13)	114.0 (97.5, 152.0)	
HAART options^b^	3TC + TDF + EFV (*N* = 118)	91.5 (54.8, 136.3)	0.872
	3TC + AZT + EFV (*N* = 27)	92.0 (40.0, 122.0)	
	NVP+3TC + AZT (*N* = 9)	93.0 (−26.5, 150.0)	
	Others (*N* = 66)	95.0 (23.3, 133.0)	
HAART replacement	Yes (*N* = 68)	83.5 (19.8, 131.8)	0.143
	No (*N* = 152)	97.0 (44.5, 136.8)	
Cr^b^	≤71.5 (*N* = 98)	83.0 (24.8, 128)	0.006
	>71.5 (*N* = 97)	113.0 (65.5, 155.0)	
	Missing (*N* = 25)	86.0 (2.0, 114.0)	
Liver function impairment^b^	Yes (*N* = 73)	85.0 (34.0, 135.5)	0.310
	No (*N* = 124)	146.8 (100.5, 165.0)	
	Missing (*N* = 23)	82.0 (−12.0, 113.0)	
Leukopenia^b^	Yes (*N* = 46)	90.0 (43.3, 123.8)	0.458
	No (*N* = 150)	98.5 (47.8, 150.3)	
	Missing (*N* = 24)	80.5 (−7.3, 111.8)	
Anemia^b^	Yes (*N* = 24)	102.0 (38.0, 135.8)	0.920
	No (*N* = 172)	96.0 (45.0, 146.0)	
	Missing (*N* = 24)	80.5 (−7.3, 128.5)	

^a^Influencers in baseline, ^b^Influencers in follow-up. Data are presented Median (IQR). *P* values were determined by the Mann–Whitney *U* test or Kruskal−Wallis test.

**Table 4:** Univariate and multivariate linear regression analyses between Influencers and CD4^+^ cell count increase.

Variable	Univariate linear regression		Multivariate linear regression	
	*β* (95% CI)	*P*	*β* (95% CI)	*P*
Gender	−13.9 (−53.3, 25.6)	0.490	−6.6 (−50.0, 36.8)	0.764
BMI	−4.1 (−8.1, −0.0)	0.048	−4.2 (−8.1, −0.2)	0.038
Age at follow-up	0.1 (−0.7, 0.8)	0.875	−0.1 (−0.9, 0.8)	0.857
Duration from HIV diagnosis to HAART start (months)^b^	−29.7 (−45.8, −13.6)	0.000	−17.0 (−33.4, −0.5)	0.043
Duration of HAART (months)^b^	−13.3 (−27.9, 1.3)	0.074	4.9 (−10.9, 20.7)	0.542
Transmission route	2.5 (−15.3, 20.3)	0.783	11.8 (−7.0, 30.6)	0.216
WHO clinical stage	24.2 (10.2, 38.1)	0.001	10.6 (−4.1, 25.2)	0.156
Co-trimoxazole^a^	−76.0 (−100.6, −51.4)	0.000	−98.5 (−129.3, −67.7)	0.000
HAART options^a^	−7.6 (−20.9, 5.7)	0.259	9.2 (−6.0, 24.3)	0.234
HAART replacement	−15.1 (−41.2, 11.0)	0.255	−19.8 (−46.5, 7.0)	0.146
Cr^b^	1.0 (0.2, 1.9)	0.013	0.9 (0.1, 1.7)	0.022

^a^Influencers in baseline, ^b^Influencers in follow-up. The multivariate linear regression model included gender, BMI, age at follow-up, duration from HIV diagnosis to HAART start, duration of HAART, transmission route, WHO clinical stage, co-trimoxazole, HAART options, HAART replacement, cr.

**Table 5:** Multivariate linear regression analyses between Influencers and CD4^+^ cell count increase, stratified analyses by BMI.

Variable	BMI < 18.5	BMI 18.5–25.0	BMI ≥ 25.0
Gender	−2.2 (−83.5, 79.0)	−6.0 (−56.3, 44.2)	−38.9 (−211.5, 133.6)
Age at follow-up	0.4 (−1.2, 2.0)	−0.4 (−1.4, 0.6)	−1.5 (−4.6, 1.6)
Duration from HIV diagnosis to HAART start (months)^b^	−24.6 (−58.5, 9.2)	−13.5 (−31.1, 4.1)	−50.7 (−138.3, 36.9)
Duration of HAART (months)^b^	−5.0 (−43.3, 33.3)	−1.6 (−18.7, 15.5)	18.5 (−41.1, 78.0)
Transmission route	1.6 (−29.5, 32.6)	13.1 (−8.7, 34.9)	−32.4 (−133.7, 68.8)
WHO clinical stage	20.0 (−8.3, 48.4)	1.3 (−15.0, 17.6)	52.8 (−8.9, 114.6)
Co-trimoxazole^a^	−93.7 (−157.8, 29.5)^*∗*^	−82.0 (−114.9, −49.1)^*∗*^	−595.7 (−756.2, −435.2)^*∗*^
HAART options^a^	18.1 (−12.5, 48.6)	6.9 (−9.7, 23.5)	28.7 (−72.1, 129.4)
HAART replacement	−54.2 (−101.2, −7.2)^*∗*^	−5.2 (−36.0, 25.6)	−65.2 (−202.0, 71.6)
Cr^b^	1.5 (0.1, 3.0)^*∗*^	0.7 (−0.2, 1.6)	−1.5 (−4.3, 1.3)

^a^Influencers in baseline, ^b^Influencers in follow-up,  ^*∗*^*P* < 0.05. The multivariate linear regression model included gender, age at follow-up, duration from HIV diagnosis to HAART start, duration of HAART, transmission route, WHO clinical stage, co-trimoxazole, HAART options, HAART replacement, cr.

## Data Availability

The dataset supporting the conclusions of this article is available by contacting the authors.

## Supplementary Materials

Supplementary Materials Table S1: characteristics in baseline and follow-up. Table S2: the comparison of follow-up and baseline clinical conditions of the study patients.
