# Supplementary material for: Analysis of the Influencing Factors of Immunological Nonresponders in Wuhan, China
Source: Can J Infect Dis Med Microbiol. 2022 Aug 8;2022:5638396. doi: 10.1155/2022/5638396 (PMC9377976; doi:10.1155/2022/5638396)
Supplement: Supplementary Materials — Table S1: characteristics in baseline and follow-up. Table S2: the comparison of follow-up and baseline clinical conditions of the study patients. [file 5638396.f1.docx]

**Table S1:** Characteristics in baseline and follow-up.

| **Baseline characteristics** | **Follow-up characteristics** |
| --- | --- |
| Age | Age |
| Gender | Gender |
| BMI | Number of follow-up |
| Transmission route | / |
| WHO clinical stage(I, II, III, IV) | / |
| Years since HIV diagnosis | Years since HAART |
| type of HAART  (3TC+TDF+EF, 3TC+AZT+EFV,  NVP+3TC+AZT, Others)  (China, 2018) | type of HAART  (3TC+TDF+EF,3TC+AZT+EFV,  NVP+3TC+AZT, Others)  (China, 2018) |
| co-trimoxazol (yes, no) | co-trimoxazol (yes, no) |
| Viral load (copies/ml) | Viral load (copies/ml) |
| CD4 counts (cells/μl) | CD4 counts (cells/μl) |
| WBC count (×10^9^ / L) | WBC count (×10^9^ / L) |
| Plt count (×10^9^ / L) | Plt count (×10^9^ / L) |
| Hb count (g/L) | Hb count (g/L) |
| Cr (μmol/L) | Cr (μmol/L) |
| TG (mmol/L) | TG (mmol/L) |
| TC (mmol/L) | TC (mmol/L) |
| GLU (mmol/L) | GLU (mmol/L) |
| AST (U/L) | AST (U/L) |
| ALT (U/L) | ALT (U/L) |
| T.BIL (μmol/L) | T.BIL (μmol/L) |

**Table S2:** The comparison of follow-up and baseline clinical conditions of the study patients.

| Variable | Baseline N（%） | | Follow-up N（%） | *p* |
| --- | --- | --- | --- | --- |
| Tuberculosis |  | |  | 0.000 |
| Yes | 21(9.5%) | | 0(0%) |  |
| No | 195(88.6%) | | 220(100%) |  |
| Unknown | 4(1.8%) | | 0(0%) |  |
| Skin damage |  | |  | 0.000 |
| Yes | 28(12.7%) | | 0(0%) |  |
| No | 192(87.3%) | | 220(100%) |  |
| Thrush |  | |  | 0.000 |
| Yes | 15(6.8%) | | 0(0%) |  |
| No | 205(93.2%) | | 220(100%) |  |
| White hair spots in the mouth |  | |  | 0.000 |
| Yes | 4(1.8%) | | 0(0%) |  |
| No | 216(98.2%) | | 220(100%) |  |
| Persistent diarrhea (> one month) |  | |  | 0.000 |
| Yes | 7(3.2%) | | 0(0%) |  |
| No | 213(96.8%) | | 220(100%) |  |
| Intermittent hair(> one month) |  | |  | 0.000 |
| Yes | 28(12.7%) | | 0(0%) |  |
| No | 192(87.3%) | | 220(100%) |  |
| Bacterial infection |  | |  | 0.000 |
| Yes | 7(3.2%) | | 0(0%) |  |
| No | 213(96.8%) | | 220(100%) |  |
| Extra-tuberculosis |  | |  | 0.000 |
| Yes | 3(1.4%) | | 0(0%) |  |
| No | 217(98.6%) | | 220(100%) |  |
| Bacterial pneumonia |  | |  | 0.000 |
| Yes | 14(6.4%） | | 0(0%) |  |
| No | 206(93.6%) | | 220(100%) |  |
| Herpes simplex virus infection |  | |  | / |
| Yes | 0(0%) | | 0(0%) |  |
| No | 220(100%) | | 220(100%) |  |
| Herpes zoster |  | |  | 0.000 |
| Yes | 12(5.5%) | | 0(0%) |  |
| No | 208(94.5%) | | 220(100%) |  |
| Other diseases |  | |  | 0.000 |
| Yes | 116(52.7%) | | 1(0.5%) |  |
| No | 104(47.3%) | 219(99.5%) | |  |

Data are presented as N(%). *p* values were determined by Wilcoxon Signed Rank Test.
